# Comparative assessment of cognitive impairment and oxidative stress markers among vitamin D insufficient elderly patients with and without type 2 diabetes mellitus (T2DM)

**DOI:** 10.1371/journal.pone.0269394

**Published:** 2022-06-16

**Authors:** Rajalakshmi R., Chinnappa A. Uthaiah, Ramya C. M., SubbaRao V. Madhunapantula, Paramahans V. Salimath, Praveen K., Srinath K. M., Kishor M. R.

**Affiliations:** 1 Department of Physiology, JSS Medical College, JSS Academy of Higher Education & Research, Mysuru, Karnataka, India; 2 Center of Excellence in Molecular Biology and Regenerative Medicine (CEMR) Laboratory (A DST-FIST Supported Center), Department of Biochemistry (A DST-FIST Supported Department), JSS Medical College, JSS Academy of Higher Education & Research, Mysuru, Karnataka, India; 3 JSS Medical College, JSS Academy of Higher Education & Research, Mysuru, Karnataka, India; 4 Department of Community Medicine, JSS Medical College, JSS Academy of Higher Education & Research, Mysuru, Karnataka, India; 5 Department of Medicine, JSS Medical College & Hospital, JSS Academy of Higher Education & Research, Mysuru, Karnataka, India; 6 Department of Psychiatry, JSS Medical College & Hospital, JSS Academy of Higher Education & Research, Mysuru, Karnataka, India; Auburn University, UNITED STATES

## Abstract

**Background:**

Disorders of mental health are known to affect cognitive functions, hence called as cognitive disorders. Impaired glucose metabolism, insulin resistance, vitamin-D deficiency and oxidative stress are some of the key early events reported to be involved in the pathogenesis of most common cognitive disorders, which include Alzheimer’s disease. Type-2 diabetes mellitus (T2DM) is one of the known contributing factors of cognitive impairment and dementia.

**Methods:**

A cross sectional study was carried out in 145 subjects, who were assessed for cognitive function by modified mini mental status examination (3MS). In addition, measurement of fasting blood sugar (FBS), fasting insulin, HbA1c, lipid profile, vitamin D and oxidative markers was performed. Participants were divided into different groups based on (a) vitamin D insufficiency and sufficiency; (b) diabetic and non-diabetic with and without cognitive impairment.

**Results:**

The study included a total of 145 subjects; 51 males and 94 females and the mean age was 68.24±9.70 years. Among ***diabetics*** with vitamin D insufficiency, 35 subjects (71.43%) had cognitive impairment, but, among ***non-diabetics*** with vitamin D insufficiency, 27 subjects (62.79%) had cognitive impairment. Chi square test showed no significant association between diabetes, vitamin D insufficiency and cognitive impairment. Glutathione peroxidase (GPx) and superoxide dismutase (SOD) levels were non-significantly lower in cognition-impaired subjects, when compared to cognition normal subjects in diabetics with vitamin D insufficiency.

**Conclusion:**

Our study showed that cognitive impairment is more predominant in individuals with diabetes. However, our study did not find any significant relationship between T2DM, vitamin D deficiency, cognitive impairment, and oxidative stress. A significant association was found only with GPx and 3MSE score in vitamin D insufficient non-diabetics.

## Introduction

Cognition is the mental process of acquiring knowledge [1]. Cognitive disorders (CDs) are a category of mental health disorders that primarily affect cognitive functions. CDs include dementia, amnesia and disorders of motor skills (what about Alzheimer’s disease [1]. Increasing life expectancy is contributing to rapid increase in prevalence of cognitive disorders in India as well as across the globe [2, 3]. According to Dementia in India Report 2020, which is published by Alzheimer’s and Related Disorders Society of India (ARDSI), about 5.3 million Indians (1 in 27 individuals) above the age of 60 years are suffering from dementia. Recent predictions projected that this number is likely to increase at an alarming rate by the year 2030 [4]. Alzheimer’s disease (AD) is the most common form of dementia. AD is a chronic neurodegenerative disease affecting the elderly people and is characterized by accumulation of amyloid beta (Aβ) peptides and neurofibrillary tangles (NFTs) [5]. Accumulating evidences have reported that impaired glucose metabolism, insulin resistance with altered insulin-signaling pathways, mitochondrial dysfunction and oxidative stress are some of the key early events reported to be involved in the pathogenesis of sporadic AD [6].

Type 2 diabetes mellitus (T2DM) is a metabolic disorder with hyperinsulinemia, insulin resistance (IR) and hyperglycemia [7]. According to global statistics by World Health Organization (WHO), diabetes is one of the biggest health concerns in the world. The level of morbidity and mortality due to diabetes and its potential complications are enormous and pose significant healthcare burden on families and society [8]. Numerous clinical and epidemiological studies have linked T2DM with an increased risk of cognitive impairment and dementia [9–11]. For instance, defect in insulin signaling is associated with decreased cognitive ability as reported in AD [12].

Vitamin D is a fat-soluble steroid like hormone with a key role in mineral metabolism, in particular, the metabolism of calcium [13]. Evidences from recent studies have linked serum vitamin D deficiency with cognitive impairment and dementia [14]. For example, many recent clinical studies have shown an increase in the risk of developing cognitive impairment only in participants with lower 25(OH)D levels [15]. Supporting this clinical observation, many *in vitro* studies have demonstrated that vitamin D can stimulate the clearance of amyloid plaques by inducing phagocytosis in macrophages thereby reduce amyloid-induced cytotoxicity, apoptosis and inflammatory responses in primary cortical neurons [16, 17]. Furthermore, a 7-year follow-up study by Annweiler et al. in 2012 showed a strong association with lower risk of developing AD among older women with elevated intake of vitamin D [18]. Vitamin D plays an important role in mitigating the development of insulin resistance there by the T2DM [19]. Mechanistically, 25-dihydroxyvitamin D inhibits the release of pro-inflammatory cytokines, tumors necrosis factor (TNF)–*α* and regulates the activity of nuclear factor (NF)-*κ*B [20, 21], while suppressing the expressions of toll-like receptors (TLR)-2 and -4 [22]. Preliminary studies have also shown that vitamin D reduces insulin resistance and the risk of diabetes by decreasing inflammatory responses [23].

Insulin resistance and oxidative stress (OS) are the common factors in T2DM and vitamin D deficiency, which can induce cognitive dysfunction [24]. OS results from an imbalance in the production and removal of reactive oxygen species (ROS). OS is an important determinant in the pathogenesis of AD as well as in the development of insulin resistance and other diabetic complications [25, 26]. Thus, this study was undertaken to determine the association between T2DM, vitamin D deficiency, cognitive impairment and oxidative stress.

## Materials and methods

This cross-sectional study (conducted in JSS Hospital) was approved by the Institutional Ethical Committee of JSS Medical College, Mysuru (JSSMC/IEC/14/3712/2016-17) for a period of 2 years beginning from 2018. By purposive sampling, patients attending outpatient department (OPD) were screened consecutively and 145 study participants in the age group of 43–90 years and meeting the inclusion and exclusion criteria were selected. The sample size was calculated at 5% level of significance and 80% power. The study participants with history of > 5 years of Type 2 DM and normal subjects without diabetes were included in the study. Those subjects with obesity, any cardiovascular diseases, other metabolic disorders, autoimmune diseases, neurological disorders and carcinomas were excluded from the study. An informed consent was obtained from all the study participants before the data collection. Informed consent forms for study participants were prepared in two languages (English and Local language) and administered to all the participants recruited under the study. These informed consent forms were approved by the Institutional Ethical Committee along with study protocol. On initial interaction with the participant it was asked whether her or herself would decide on study participation or required presence of either legally authorized representative (LAR) or impartial witness from the study participants. The decision was taken on consent process accordingly. In case of participant was not able to answer this question, consent was done in presence of LAR only. A trained physician carried out clinical examination and collected detailed medical history from each study participant.

### Collection of blood sample

A phlebotomist collected 6.0 ml of blood in the fasting state by venipuncture and transferred in to an EDTA containing blood collection tube (BD Vaccutainers, Catalog no:367863) for the analysis of glycated haemoglobin (HbA1c), and haemoglobin percentage (Hb). But, for the estimation of vitamin-D, fasting serum insulin (FSI), fasting blood sugar (FBS) and other biochemical parameters samples were collected into the tubes containing clot activators (BD Vaccutainers, Catalog no:368815). The serum was separated by centrifugation at 4000 rpm for 15 minutes and aliquoted for storage at—80°C until further use.

### Cognition assessment

All recruited study participants were assessed for cognitive function using modified mini mental status (3MS) test [27, 28]. The 3MS test is a widely used validated standard neuropsychological test not only for assessing the cognitive function among the elderly, but also for screening individuals for Alzheimer’s disease and other causes of dementia. It includes tests of orientation, registration, attention, calculation, recall, and visual spatial skills. Objective scoring of 3MS test ranged from 0–100. Cognition is said to be impaired, if the score is less than 80 out of 100 in the 3MS test [27, 28]. Those who had low cognitive score were subjected to detailed mental examination and subjects were classified into two groups with and without cognitive deficit.

### Study groups

**Fig 1:**
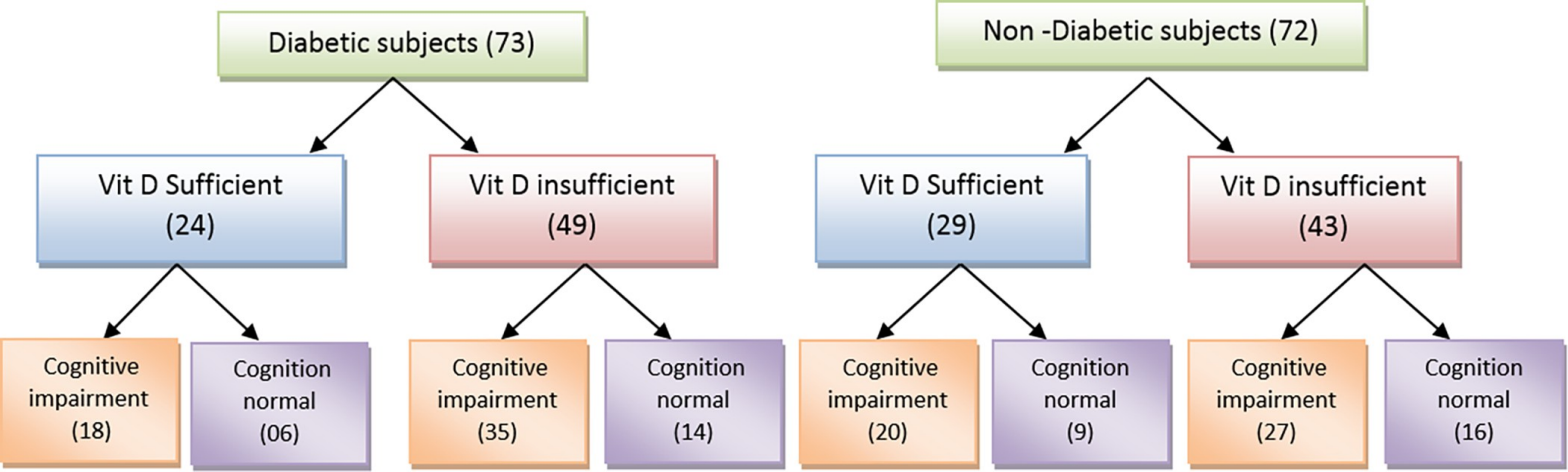
Schematic flow chart for recruitment and classification of study subjects based on the diabetes condition, vitamin-D levels and cognition status (3MSE).

Based on medical history and HbA1c level, the study subjects were grouped as diabetics and non-diabetics. These study groups were further categorized in to (a) vitamin-D sufficient; and (b) vitamin D insufficient based on vitamin D levels. The participants with vitamin D level ≤ 29 ng/mL were considered as vitamin D insufficient and those with ≥ 30 ng/mL were considered as vitamin D sufficient [29]. Further classification was performed based on 3MS score of the study participants into the one with cognitive impairment and the one without cognitive impairment as shown in flow chart **(Fig 1)**.

### Measurement of FBS, fasting insulin and HbA1c

The fasting blood sugar (FBS) was measured in freshly collected samples by Cobas c 501 (Roche, USA) using Gluc3-Glucose hexokinase kit (Catalogue no: 04404483–190) from Roche, USA.

The fasting serum insulin level was measured freshly collected samples by fully automated electro immunoassay analyzer (Cobas e 601 from Roche, USA) using Elecsys Insulin kit (Catalogue no: 12017547–122, from Roche, USA).

The level of HbA1c was estimated in freshly collected samples by Bio-Rad D-10 haemoglobin system (Bio-Rad, USA) using D-10 Hemoglobin A1c kit (Catalogue no: 220–0101, from Bio-Rad, USA).

Homeostatic model assessment-insulin resistance (HOMA-IR) was calculated using FBS and fasting insulin level.

### Measurement of lipid profile

The lipid profile was measured in freshly collected samples by fully automatic analyzer (Cobas c 501, Roche, USA). TRIGL triglycerides kit (Catalogue no: 20767107–322), HDL3 HDL-Cholesterol plus 3rd generation kit (Catalogue no: 04399803–190), LDLC3 LDL-Cholesterol Gen.3 kit (Catalogue no: 07005717–190) and CHOL2 Cholesterol Gen.2 kit (Catalogue no: 03039773–190) from Roche, USA were used to determine the level of triglycerides, HDL, LDL, and cholesterol, respectively.

### Measurement of vitamin D levels

Vitamin D level was measured in the freshly separated serum by fully automatic electro-immunoassay analyzer (Cobas e 601,) Roche, USA) using vitamin D total elecsys kit (Catalogue no: 05894913–190,) from Roche, USA).

### Measurement of superoxide dismutase (SOD), glutathione peroxidase (GPx) and total antioxidant activity in the serum

The activity of SOD and GPx was determined by thawing the separated serum, which was stored at -80°C using commercially available high throughput kit from R&D systems (Catalogue no: 7501-500-K, Catalogue no: 7512-100-K respectively for SOD and GPx) The samples were thawed only once to perform enzyme assays and total antioxidant activity estimation. The total antioxidant activity, which is measuring the thiobarbituric acid reacting substances (TBARS), was estimated according to Koracevic et al., 2001. Briefly, 10μL of serum was made up to 0.5mL with sodium phosphate buffer (100mM, pH-7.4) and reaction mixture was prepared by adding 0.5mL sodium benzoate (10mM), 0.2mL of ferrous ammonium sulphate-EDTA complex (Prepared by mixing Ferrous ammonium sulphate-2mM and EDTA-2mM at 1:1 ratio), 0.2mL of hydrogen peroxide (10mM) and incubated the mixture for 60 minutes at room temperature in dark. The mixture was added with 1.0mL of acetic acid (20%), 1.0mL of thiobarbituric acid (0.8% in 50mM NaOH) and kept for incubation at boiling water bath for 10 minutes, the tubes were cooled and pale pink colour developed was measured at 532nm against water as blank. The total antioxidant activity in the samples was calculated based on inhibiting the formation of TBARS and representing the data in percentage inhibition. Standards containing uric acid ranging from 0.156 to 0.625mM were used for constructing a calibration graph. For each series of analysis a negative control was prepared, containing the same reagents as added to that of samples, except that serum is replaced with phosphate buffer. For each sample, a respective blank (sample blank) was also prepared to estimate background level due to the substances that may interfere with the reaction [30].

### Statistical analysis

Data collected was entered in MS-Excel-2010 and analyzed using statistical package for social sciences (SPSS) version 23.0 licensed to the institution. Descriptive statistical measures like percentage, mean and standard deviation were applied. Inferential statistical tests like chi square/Fisher’s exact probability test were applied to determine the association between categorical variables. Independent sample test was applied to assess the difference between mean values across different groups. Pearson’s and Spearman correlation were applied to assess correlation between continuous variables. Association, difference and correlation tests were considered statistically significant if p<0.05.

## Results

The study, which included a total of 145 subjects, had 51 males (35.2%) and 94 females (64.8%). The mean age of study participants was 68.24±9.70 years. Among study participants 92 (63.45%) had vitamin D insufficiency and 100 (69%) had cognitive impairment. Study subjects were divided into diabetics (n = 73, 50.3%) and non-diabetics (n = 72, 49.7%) based on their HbA1c value. The characteristics of study participants were depicted in **Table 1**, which showed a statistically significant difference (P < 0.05) between non-diabetic and diabetic groups in FBS, fasting serum insulin, HOMA IR, HbA1c, triglycerides, HDL, LDL and VLDL levels. No significant difference was found in the mean value of vitamin D and 3MSE score between the groups. SOD levels were lower in diabetic group, but not statistically significant. TBARS and GPx levels showed minimal variation between the groups.

**Table 1:** Comparison of study participant characteristics between diabetic and non-diabetic groups.

Characteristic	Diabetics (n = 73)	Non-diabetics (n = 72)	P value
	Mean ± SD	Mean ± SD	
Age (years)	69.07 ± 9.64	67.40 ± 9.75	0.302
Gender	M: F (29:43)	M: F (22:51)	
BMI			
FBS (mg/dL) (70 to 100 mg/dL)	123.26 ± 68.43	81.62 ±23.30	0.001**
FSI (mIU/L) (2 to 25 mIU/L)	9.39 ± 5.87	6.65 ± 3.89	0.001**
HOMA-IR (0.5 to 2.6)	2.75 ± 2.15	1.39 ±0.98	0.001**
HbA1c % (< 5.7%)	7.89 ± 2.22	5.78 ±0.39	0.001**
Haemoglobin (g%) (12 to 16 g%)	12.91 ± 1.54	12.48 ± 2.75	0.257
Vitamin D (ng/mL) (30 to 50 ng/mL)	27.45 ± 9.70	27.68 ± 10.12	0.889
3MSE Score (81–100)	59.38 ± 24.33	58.50 ± 25.97	0.833
Total cholesterol (mg/dL) (<200 mg/dL)	174.08 ± 39.25	179.71 ± 35.11	0.365
HDL (mg/dL) (> 60 mg/dL)	41.71 ± 7.67	45.22 ± 8.17	0.009**
LDL (mg/dL) (<130 mg/dL)	99.110 ± 28.06	109.74 ± 23.87	0.015*
VLDL (mg/dL) (<30 mg/dL)	31.59 ± 14.46	24.42 ± 9.99	0.001**
Triglycerides (mg/dL) (<150 mg/dL)	158.56 ± 72.34	123.13 ± 49.72	0.001**

### More cognitive impairment was observed in individuals with diabetes and vitamin D insufficiency

**Fig 2:**
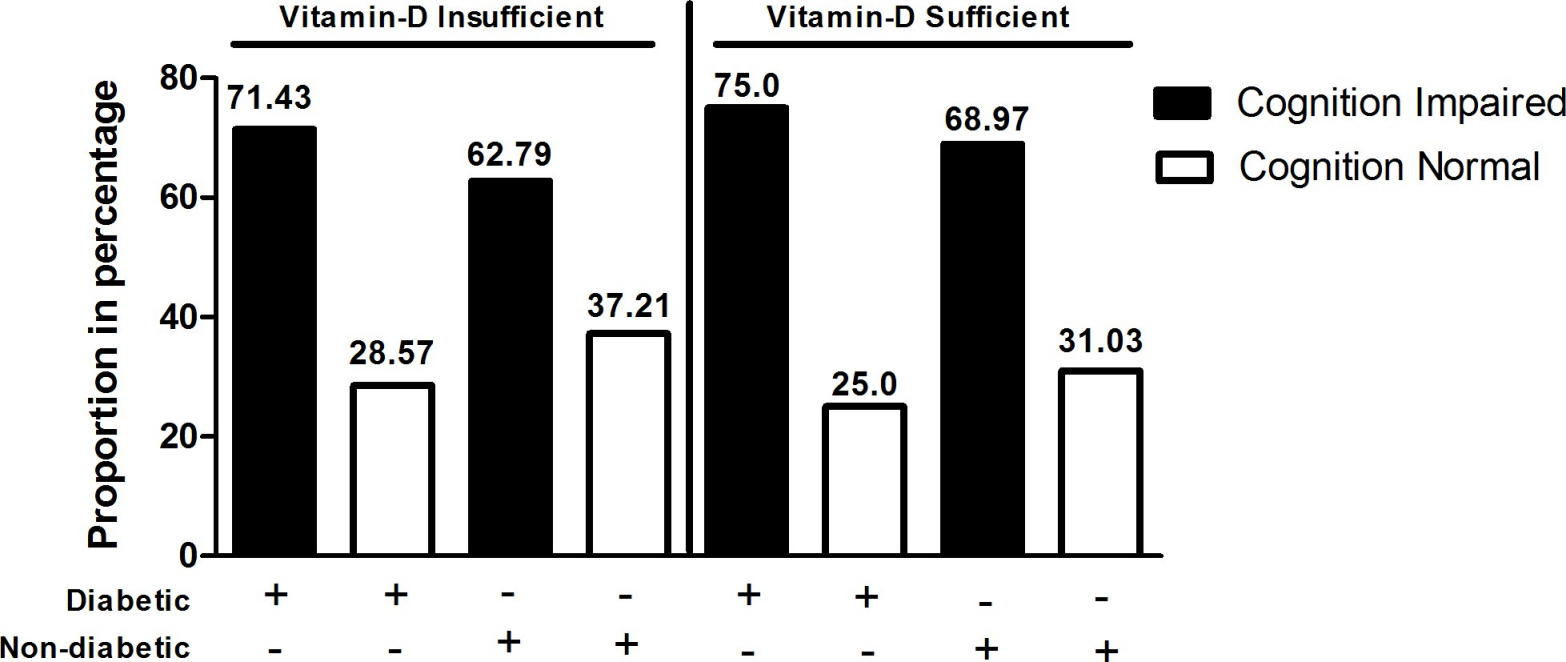
Percentage proportion of subjects in each group represents diabetic (71.43%) and non-diabetic (62.79%) subjects having vitamin-D insufficiency with cognition impairment whereas diabetic (75%) and non-diabetic (68.97%) were vitamin-D sufficient subjects with cognition impairment.

To determine whether diabetes and vitamin D status of individuals have any impact on cognitive impairment status, first, we have quantified the vitamin D in the serum of study participants as detailed in materials and methods and the results were compared between diabetic and non-diabetic groups **(Fig 2)**. Analysis of the data showed among diabetics with vitamin D insufficiency, 35 subjects (71.43%) had cognitive impairment, but, among diabetics with sufficient vitamin D level, 18 subjects (75%) had cognitive impairment. Among non-diabetics with vitamin D insufficiency, 27 subjects (62.79%) had cognitive impairment and among non-diabetics with vitamin D sufficiency, 20 subjects, (68.97%) had cognitive impairment. Chi square test showed no significant association between diabetes and vitamin D insufficiency, and cognitive impairment.

**Fig 3:**
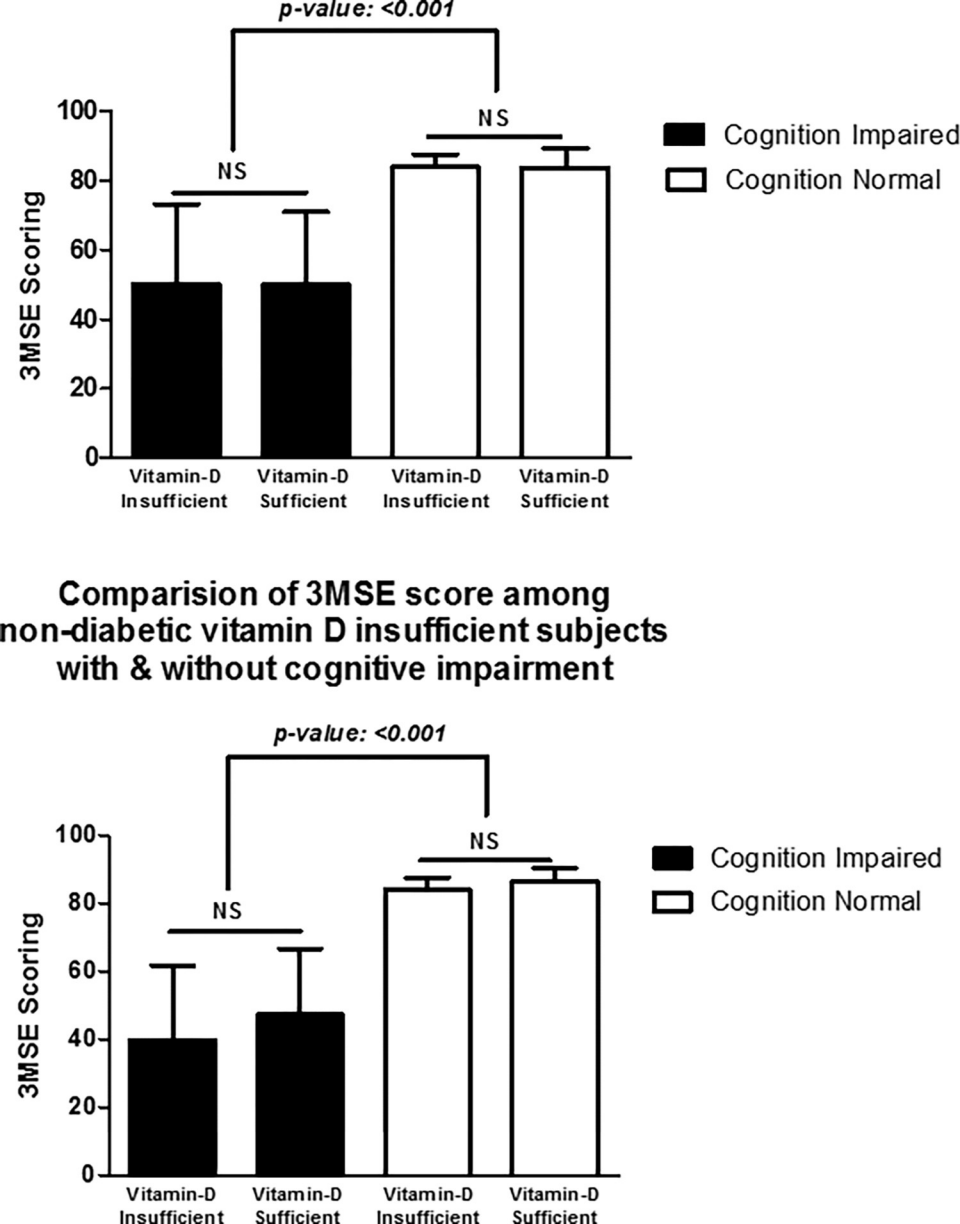
Comparison of 3MSE scoring in diabetic and non-diabetic subjects among vitamin-D insufficiency and sufficiency group.

The biochemical parameters of the diabetics with vitamin D insufficiency with and without cognitive impairment is shown in **Table 2**. Among diabetics with vitamin D insufficiency, a statistically significant (P<0.05) difference in the 3MS score was observed between cognitive impairment and cognition normal groups **(Fig 3)**. The mean value of 3MS score was 50.20 ± 23.08 and 83.93 ± 3.66 between cognitive impairment and cognition normal among diabetics with vitamin D insufficiency, respectively, which was statistically significant. Similarly, among diabetics with vitamin D sufficiency, a statistically significant (P value < 0.05) difference in the 3MS score was observed between cognitive impairment and cognitive normal groups. The mean value of 3MS score were 50.00 ± 21.16 and 83.83 ± 5.56 between cognitive impairment and cognition normal, among diabetics with vitamin D sufficiency, respectively, which was statistically significant. The mean values of vitamin D were 22.18 ±4.80 and 21.96 ±5.91 between cognitive impairment and cognition normal among diabetics with vitamin D insufficiency, respectively, which was statistically not significant. However, in diabetics with vitamin D sufficiency the mean 3MS score was 36.50 ±4.60 and 43.86 ±11.81, between cognitive impairment and cognitive normal and was statistically significant.

**Table 2:** Comparison of various parameters among diabetic subjects with vitamin D insufficiency and cognitive impairment.

Parameter	Vitamin D Insufficiency			Vitamin D sufficiency		
	Cognitive impairment	Cognition Normal	P Value	Cognitive impairment	Cognition Normal	P Value
FBS (mg/dl)	129.21 ±71.80	115.81 ±76.79	0.566	122.65 ±65.47	107.71 ±42.20	0.608
FSI 2 to 25 (mIU/L)	10.85 ±7.21	9.59 ±4.16	0.541	7.06 ±4.07	7.46 ±1.68	0.823
HOMA-IR	3.07 ±2.06	3.21 ±3.30	0.863	1.99 ±1.12	2.03 ±1.16	0.940
HbA1c %	8.40 ±2.40	7.24 ±2.39	0.132	7.85 ±1.82	6.60 ±0.86	0.037
Haemoglobin (gm %)	12.81 ±1.62	12.80 ± 1.50	0.978	13.11 ±1.44	13.10 ±1.72	0.982
Vitamin D (ng/mL)	22.18 ±4.80	21.96 ±5.91	0.893	36.50 ±4.60	43.86 ±11.81	0.035*
3MSE Score	50.20 ± 23.08	83.93 ± 3.66	0.001*	50.00 ± 21.16	83.83 ± 5.56	0.001*
Total cholesterol (mg/dL)	181.00 ±36.80	178.79 ±44.43	0.859	159.72 ±36.71	165.83 ±45.13	0.772
HDL (mg/dL)	42.31 ±7.16	40.93 ±6.24	0.530	39.89 ±9.46	45.50 ±7.79	0.205
LDL (mg/dL)	102.07 ±23.96	105.30 ±30.74	0.697	88.68 ±31.50	98.63 ±32.27	0.512
VLDL (mg/dL)	32.99 ±16.08	31.48 ±14.24	0.761	32.86 ±11.7	19.86 ±8.58	0.013
Triglycerides (mg/dL)	165.23 ±80.53	158.86 ±70.90	0.797	164.72 ±58.62	100.50 ±44.88	0.017

The clinical parameters of the non-diabetics with vitamin D insufficiency with and without cognitive impairment were given in **Table 3**. The mean values of 3MS score were 39.90 ± 21.88 and 84.00 ± 3.60 between cognitive impairment and cognition normal among non-diabetics with vitamin D insufficiency, respectively, which was statistically significant. The mean values of 3MS score were 47.26 ± 19.46 and 86.38 ± 3.94 between cognitive impairment and cognition normal among non-diabetics with vitamin D sufficiency, respectively, which was statistically significant **(Fig 3)**. The mean values of vitamin D were 22.66 ± 5.64 and 19.16 ± 7.79 between cognitive impairment and cognition normal among non-diabetics with vitamin D insufficiency, respectively, which was statistically not significant. However, in non-diabetics with vitamin D sufficiency the mean value was 35.80 ± 5.76 and 39.86 ± 7.14 between cognitive impairment and cognition normal and was not significant.

**Table 3:** Comparison of various parameters among non-diabetic subjects with vitamin D insufficiency and cognitive impairment.

Parameters	Vitamin D Insufficiency			Vitamin D sufficiency		
	Cognitive impairment	Cognition Normal	P Value	Cognitive impairment	Cognition Normal	P Value
FBS (mg/dl)	87.03 ± 19.01	74.24 ±25.93	0.070	80.30 ±27.23	81.42 ±20.28	0.913
FSI 2 to 25 (mIU/L)	8.07 ±4.35	6.21 ±4.04	0.172	5.22 ±2.87	6.40 ±3.14	0.329
HOMA-IR	1.80 ± 1.14	1.14 ± 0.92	0.047	1.07 ±0.73	1.33 ±0.66	0.371
HbA1c %	5.81 ±0.46	5.77 ±0.30	0.745	5.72 ±0.41	5.88 ±0.25	0.358
Haemoglobin (gm %)	12.09 ±3.52	13.13 ±2.64	0.316	12.43 ±2.18	12.61 ±1.39	0.822
Vitamin D (ng/mL)	22.66 ±5.64	19.16 ±7.79	0.095	35.80 ±5.76	39.86 ±7.14	0.115
3MSE Score	39.90 ± 21.88	84.00 ± 3.60	0.001**	47.26 ± 19.46	86.38 ± 3.94	0.001**
Total cholesterol (mg/dL)	170.74 ±32.22	195.13 ±25.63	0.010*	174.00 ±39.89	191.89 ±39.48	0.272
HDL (mg/dL)	43.07 ±6.73	50.75 ± 6.80	0.001**	40.65 ±7.49	52.00 ±6.72	0.001**
LDL (mg/dL)	103.54 ± 21.32	121.27 ± 15.04	0.008**	107.77 ±26.79	112.22 ±29.92	0.693
VLDL (mg/dL)	26.19 ±11.49	21.85 ±6.49	0.175	23.88 ±10.05	24.88 ±10.62	0.808
Triglycerides (mg/dL)	131.67 ±57.40	111.06 ±32.20	0.196	120.15 ±50.09	125.56 ±52.51	0.793

### Co-relation between diabetes, vitamin D insufficiency, cognitive impairment and oxidative stress markers

To determine, whether oxidative marker levels of individuals have any association with cognitive impairment status, among diabetics and non-diabetics with and without vitamin D deficiency, we have quantitated the oxidative markers in the serum of study participants as detailed in materials and methods and the results were compared between vitamin D insufficient and vitamin D sufficient diabetics and non-diabetics groups with and without cognitive impairment.

**Fig 4:**
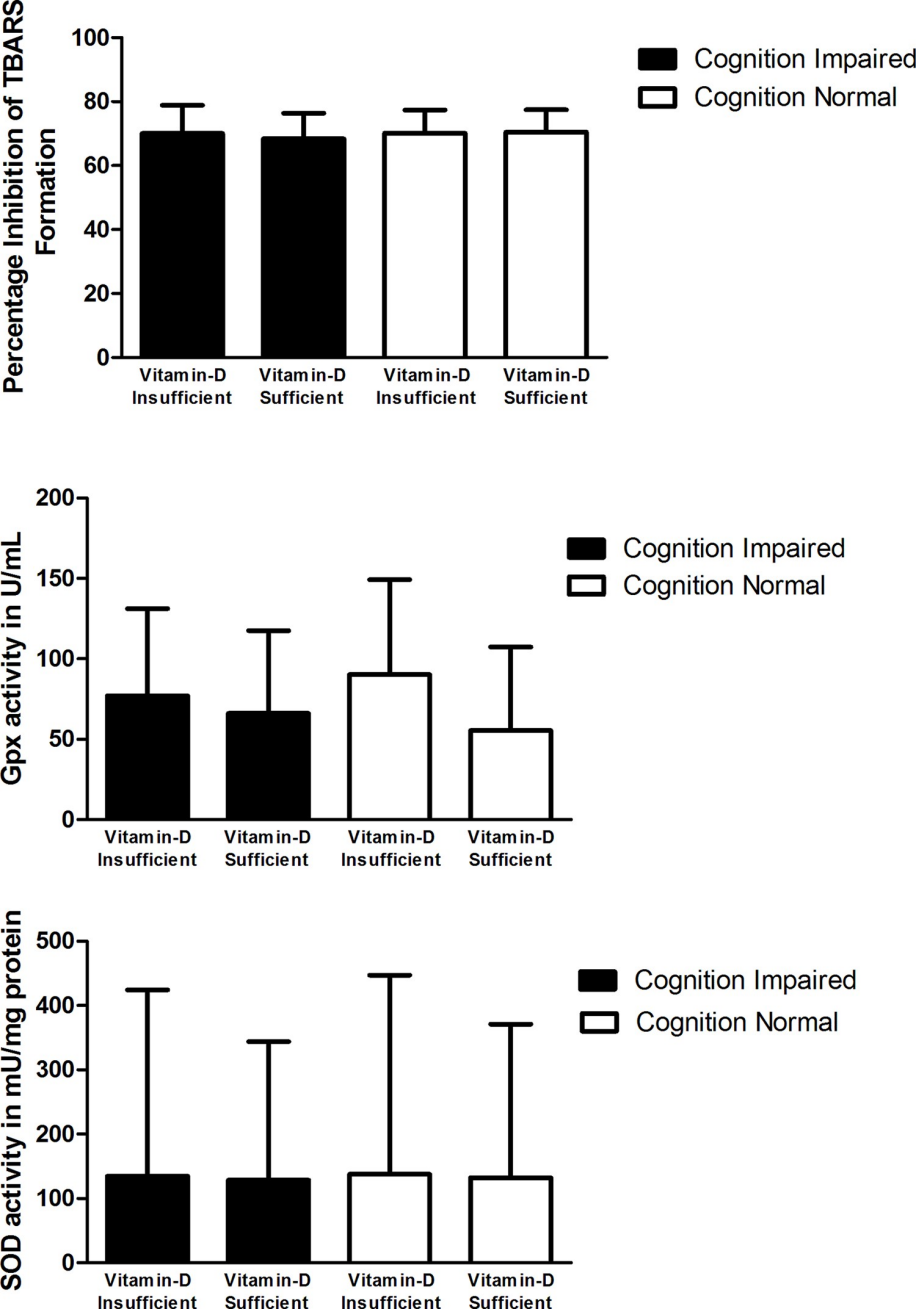
A. Comparison of total antioxidant activity by TBARS in diabetic subjects with vitamin-D and cognition status, B. Comparison of glutathione peroxidase (Gpx) activity in diabetic subjects with vitamin-D and cognition status, C. Comparison of superoxide dismutase (SOD) activity in diabetic subjects with vitamin-D and cognition status.

Among diabetics (**Table 4**):

- No difference was found in the TBARS activity between cognitive impaired and cognition normal diabetics with vitamin D insufficiency. Whereas, in cognitive impaired diabetics with vitamin D sufficiency the TBARS activity was slightly lower, which was not significant (**Fig 4A**).
- GPx levels were non significantly lower in cognition impaired subjects, when compared to cognition normal subjects in diabetics with vitamin D insufficiency (Mean levels: 77.08 ±54.24 and 90.32 ±58.86, respectively), whereas in diabetics with vitamin D sufficiency the GPx level was non-significantly higher in cognition impaired subjects, when compared to cognition normal subjects (Mean levels: 66.01 ±51.56 and 55.36 ±52.02, respectively) (**Fig 4B**).
- Similarly, SOD level was slightly lower in cognition impaired subjects when compared to cognition normal subjects in diabetics with vitamin D insufficiency (Mean levels: 134.86 ± 289.41 and 137.67 ± 309.62, respectively). In diabetics with vitamin D sufficiency also the SOD levels were non-significantly lower in cognition-impaired subjects when compared to cognition normal subjects (Mean levels: 128.44 ±215.42 and 132.42 ± 238.44, respectively) (**Fig 4C**).

**Table 4:** Comparison of oxidative markers among diabetic subjects with vitamin D insufficiency and cognitive impairment.

Parameter	Vitamin D Insufficiency			Vitamin D sufficiency		
	Cognitive impairment	Cognition Normal	P Value	Cognitive impairment	Cognition Normal	P Value
TBARS (% Inhibition)	70.03 ±8.86	70.09 ±7.25	0.982	68.36 ±8.05	70.42 ±7.04	0.584
GPX (Units/mL)	77.08 ±54.24	90.32 ±58.86	0.455	66.01 ±51.56	55.36 ±52.02	0.666
SOD (Units/mg protein)	134.86 ±289.41	137.67 ±309.62	0.976	128.44 ±215.42	132.42 ±238.44	0.366

**Fig 5:**
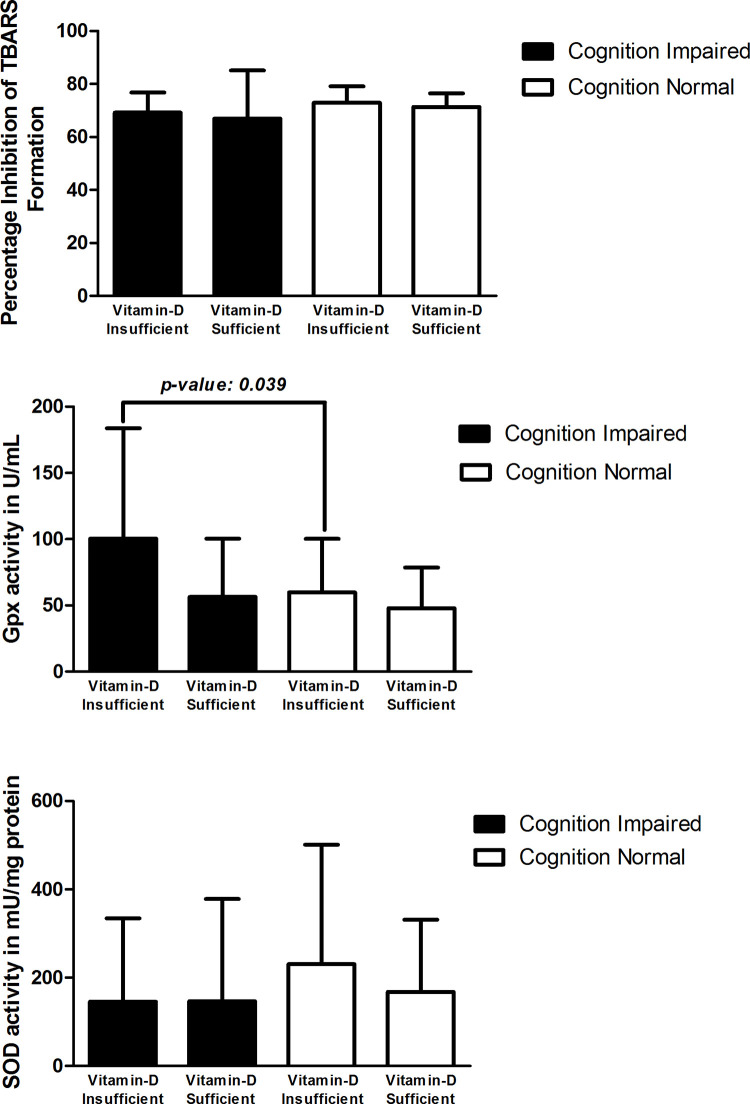
A. Comparison of total antioxidant activity by TBARS in non-diabetic subjects with vitamin-D and cognition status, B. Comparison of glutathione peroxidase (GPx) activity in non-diabetic subjects with vitamin-D and cognition status, C. Comparison of superoxide dismutase (SOD) activity in non-diabetic subjects with vitamin-D and cognition status.

Among Non-diabetics (**Table 5**):

- No difference was found in the mean value of TBARS activity and was slightly lower in both cognitive impaired and cognition normal subjects in both non-diabetics with vitamin D insufficiency and vitamin D sufficiency subjects (**Fig 5A**).
- GPx levels were significantly higher in cognition impaired subjects, when compared to cognition normal subjects in non-diabetics with vitamin D insufficiency (Mean levels:100.37±83.33 and 59.87±40.32, respectively), whereas in non-diabetics with vitamin D sufficiency the GPx levels were non-significantly higher in cognition impaired subjects, when compared to cognition normal subjects (**Fig 5B**).
- SOD levels were non-significantly lower in cognition impaired subjects, when compared to cognition normal subjects in both non-diabetics with vitamin D insufficiency and vitamin D sufficiency subjects (**Fig 5C**).

**Table 5:** Comparison of oxidative stress markers among non-diabetic subjects with vitamin D insufficiency and cognitive impairment.

Parameters	Vitamin D Insufficiency			Vitamin D sufficiency		
	Cognitive impairment	Cognition Normal	P Value	Cognitive impairment	Cognition Normal	P Value
TBARS (% Inhibition)	69.23 ±7.59	72.98 ±6.13	0.101	66.92 ±18.19	71.37 ±5.06	0.481
GPX (Units/mL)	100.37 ±83.33	59.87 ±40.32	0.039*	56.49 ±43.96	47.96 ±30.62	0.604
SOD (Units/mg protein)	145.72 ±188.49	230.59 ±270.47	0.233	146.72 ±231.59	167.94 ±163.19	0.806

**Fig 6:**
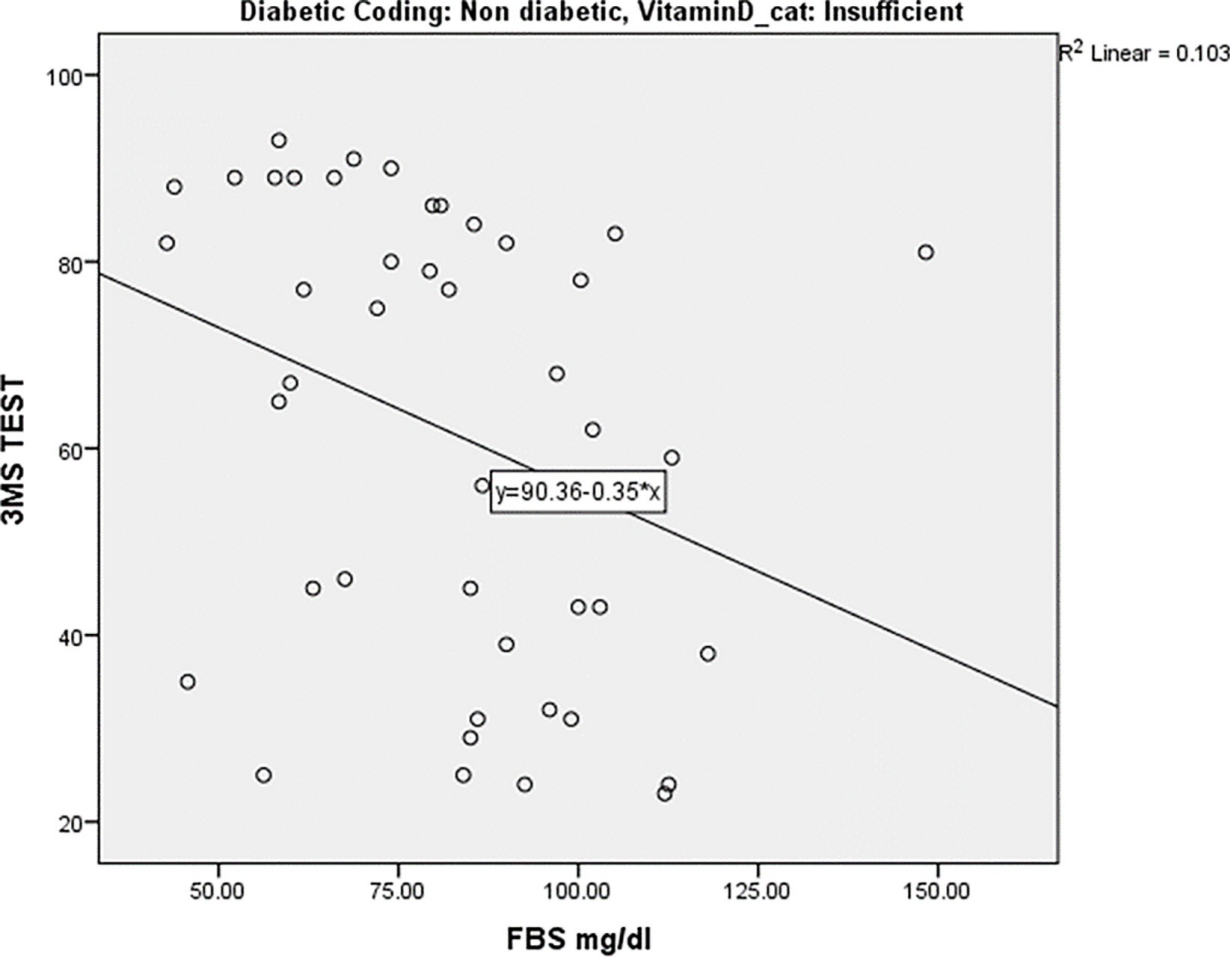
Correlation between 3MSE score and FBS in non-diabetics with vitamin D insufficiency.

**Fig 7:**
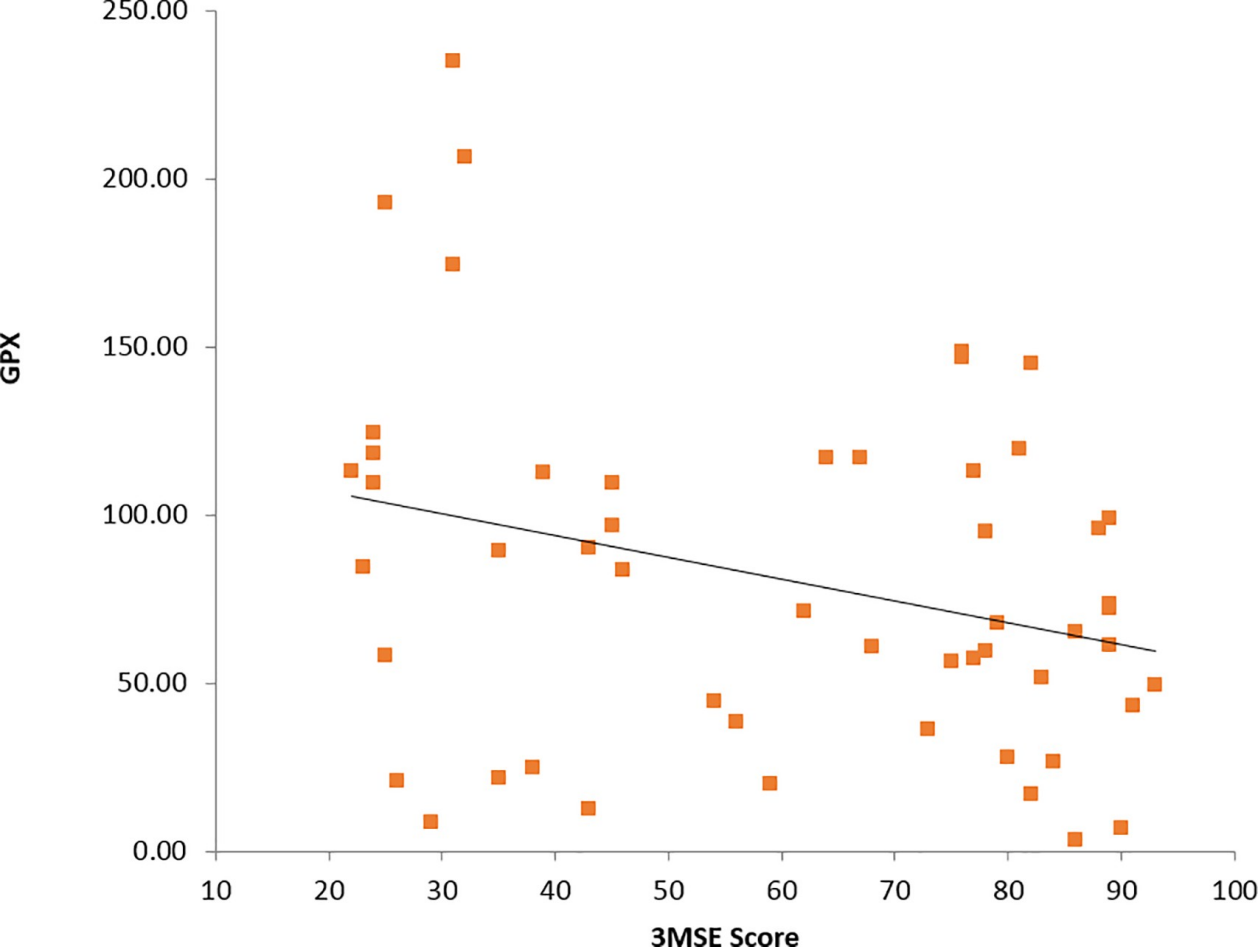
Correlation between 3MSE score and GPx in non-diabetics with vitamin D insufficiency.

Spearmann’s test was applied to know the correlation between 3MS Score and diabetic profile, vitamin D and oxidative markers, among diabetics and non-diabetics with vitamin D insufficiency and vitamin D sufficiency. The data showed: (a) no correlation among diabetics (**Tables 6 & 7**), (b) a statistically significant negative correlation between 3MS Score and FBS (R = -0.323) **(Fig 6)**, GPx (R = -0.384) in vitamin D in-sufficiency non-diabetics (**Table 8**, **Fig 7**) and (c) no correlation in vitamin D sufficiency subjects (**Table 9**).

**Table 6:** Correlation between 3MS score and various biochemical parameters among diabetic subjects with vitamin D insufficiency.

Parameters	R	P Value
FBS (mg/dl)	-0.126	0.387
Fasting insulin (mIU/L)	-0.035	0.809
HOMA-IR	0.046	0.753
HbA1c (%)	-0.176	0.225
Vitamin D (ng/ml)	-0.010	0.945
TBARS	0.058	0.690
Gpx	0.011	0.938
SOD	-0.112	0.442

**Table 7:** Correlation between 3MS score and various biochemical parameters among diabetic subjects with vitamin D sufficiency.

Parameters	R	P Value
FBS (mg/dl)	0.103	0.633
Fasting insulin (mIU/L)	0.146	0.497
HOMA-IR	0.176	0.412
HbA1c (%)	-0.116	0.590
Vitamin D (ng/ml)	0.320	0.127
TBARS	0.039	0.856
Gpx	-0.197	0.357
SOD	-0.349	0.095

**Table 8:** Correlation between 3MS score and various biochemical parameters among non-diabetic subjects with vitamin D insufficiency.

Parameters	R	P Value
FBS (mg/dL)	-0.323*	0.035
Fasting insulin (mIU/L)	-0.057	0.716
HOMA-IR	-0.184	0.238
HbA1c (%)	-0.035	0.822
Vitamin D (ng/mL)	-0.205	0.188
TBARS	0.206	0.184
GPx	-0.384*	0.011
SOD	0.288	0.061

**Table 9:** Correlation between 3MS score and various biochemical parameters among non-diabetic subjects with vitamin D sufficiency.

Parameters	R	P value
FBS (mg/dL)	0.023	0.905
Fasting insulin (mIU/L)	0.161	0.403
HOMA-IR	0.147	0.446
HbA1c (%)	0.204	0.289
Vitamin D (ng/mL)	0.155	0.422
TBARS	0.298	0.116
GPx	-0.101	0.603
SOD	-0.056	0.771

## Discussion

Vitamin D receptors are found in the central nervous system and vitamin D may provide neuroprotective effect as it influences neurogenesis, calcium signaling, the expression of neurotrophic factors and amyloid-beta clearance and help prevent neurodegeneration, which is a major cause of dementia and cognitive dysfunction [16, 31, 32]. The effect of vitamin D deficiency on T2DM and cognitive impairment, the interactions among them have recently been the subject of clinical research. Thus, the present study was conducted to investigate the association between T2DM, vitamin D deficiency, cognitive dysfunction and oxidative stress. The results of this study showed that proportion of vitamin D insufficient diabetic subjects with cognitive impairment was higher, when compared to vitamin D insufficient non-diabetics. But, vitamin D insufficient diabetics with cognitive impairment had slightly higher 3MSE scores than vitamin D insufficient non-diabetics with cognitive impairment, which was insignificant. No significant association was found between diabetes, vitamin D insufficiency and cognitive impairment. Similar to our study, Yegin et al also did not find any significant relationship between the vitamin D deficiency level and the MMSE score [33]. Contrarily, few of the previous clinical studies have shown an association between diabetes mellitus, vitamin D and cognitive dysfunctions and an increased risk of vascular dementia and Alzheimer’s disease in vitamin D deficient patients. A case control study by Parveen et al., and a cross-sectional study by Chen et al., have reported an inverse association of 25(OH)D and cognitive impairment in T2DM patients [34, 35]. A randomized control trial by Byrn et al, found, no significant differences in cognitive outcomes between T2DM patients who received high-dose therapy and those who received low dose of vitamin D. But the study did not contain a true placebo group [36]. Dean et al., also reported no significant improvements in cognitive functioning in the group receiving vitamin D supplementation compared to the group receiving placebo among young adults [37].

Vitamin D has an antioxidant effect through the inhibition of formation of free radicals [38, 39]. More over, aging, T2DM and vitamin D deficiency are associated with oxidative stress. Our results revealed that there is no correlation between oxidative markers and 3MSE score among vitamin D insufficient diabetics. A significant association was found only with GPx and 3MSE score r = -0.384 & p<0.05 in vitamin D deficient non-diabetics. A cross sectional study assessed the serum 25(OH) D levels, and its association with oxidative stress markers in T2DM and concluded that, lower levels of vitamin D is associated with increased oxidative stress [39]. Onno et al., have reported that elevated circulating biomarkers of oxidation and endothelial functions were associated with worse performance on cognitive domains that depend on global information processing in T2DM [40]. A double-blind, randomized, placebo-controlled trial in China, by Yang et al., has revealed that vitamin D supplementation for 12 months appears to improve cognitive function through reducing oxidative stress regulated by increased telomere length (TL) in older adults with mild cognitive impairment [41]. Many studies have also suggested that Vitamin D status could alter the balance between pro- and anti-inflammatory cytokines by its immuno-modulatory functions, which can affect cytokines release such as tumor necrosis factor-α (TNF-α), IL-6 and IL-12 from peripheral mononuclear cells, which in turn can induce CNS neuro-inflammation and cognitive dysfunction. In this study we have not studied the role of inflammatory markers, however, we have assessed the role of oxidative stress and the markers that represent oxidative stress [42, 43]. Although oxidative stress in the brain is linked with cognitive impairment and neuroinflammation, the anti-oxidative parameters in the peripheral circulation such as the levels of SOD, GPx and total anti-oxidative activity of serum are hardly reflective of the situations in the brain. Thus, the measuring these parameters in serum in the current study may not have shown significant results.

### Limitations

One of the limitations of the study was its sample size, which is due to existing COVID-19 situation. In addition, the seasonal variations that could impact the results are not studied in this report.

## Conclusion

In summary, our study showed that cognitive impairment is more predominant in individuals with diabetes. The study investigated, whether there is an association between T2DM, vitamin D deficiency, cognitive impairment, and oxidative stress, but did not demonstrate any significant relationship. No correlation between oxidative markers and 3MSE score among vitamin D insufficient diabetics was observed. A significant association was found only with GPx and 3MSE score in vitamin D deficient non-diabetics. A large-scale study is warranted to understand the association between T2DM, vitamin D deficiency, cognitive impairment, and oxidative stress.

## Supporting information

S1 Data (XLSX)
